# Serum Superoxide Dismutase Is Associated with Vascular Structure and Function in Hypertensive and Diabetic Patients

**DOI:** 10.1155/2016/9124676

**Published:** 2015-11-09

**Authors:** Manuel A. Gómez-Marcos, Ana M. Blázquez-Medela, Luis Gamella-Pozuelo, José I. Recio-Rodriguez, Luis García-Ortiz, Carlos Martínez-Salgado

**Affiliations:** ^1^Research Unit, Primary Care Centre La Alamedilla, SACYL, Avenida de los Comuneros 27, 37003 Salamanca, Spain; ^2^Institute of Biomedical Research of Salamanca (IBSAL), Renal and Cardiovascular Pathophysiology Unit, Department of Physiology and Pharmacology, University of Salamanca, Avenida Campo Charro s/n, 37007 Salamanca, Spain; ^3^Institute of Studies in Health Sciences of Castilla y León (IECSCYL), Research Unit, University Hospital of Salamanca, Paseo San Vicente 58-182, 37007 Salamanca, Spain

## Abstract

Oxidative stress is associated with cardiac and vascular defects leading to hypertension and atherosclerosis, being superoxide dismutase (SOD) one of the main intracellular antioxidant defence mechanisms. Although several parameters of vascular function and structure have a predictive value for cardiovascular morbidity-mortality in hypertensive patients, there are no studies on the involvement of SOD serum levels with these vascular parameters. Thus, we assessed if SOD serum levels are correlated with parameters of vascular function and structure and with cardiovascular risk in hypertensive and type 2 diabetic patients. We enrolled 255 consecutive hypertensive and diabetic patients and 52 nondiabetic and nonhypertensive controls. SOD levels were measured with an enzyme-linked immunosorbent assay kit. Vascular function and structure were evaluated by pulse wave velocity, augmentation index, ambulatory arterial stiffness index, and carotid intima-media thickness. We detected negative correlations between SOD and pressure wave velocity, peripheral and central augmentation index and ambulatory arterial stiffness index, pulse pressure, and plasma HDL-cholesterol, as well as positive correlations between SOD and plasma uric acid and triglycerides. Our study shows that SOD is a marker of cardiovascular alterations in hypertensive and diabetic patients, since changes in its serum levels are correlated with alterations in vascular structure and function.

## 1. Introduction

Hypertension is quantitatively the most important risk factor for premature cardiovascular disease; essential hypertension and diabetes are characterized by endothelial dysfunction mediated by an impaired NO availability secondary to oxidative stress production [1]. Vascular disease is one of the main causes for disability and death in patients with diabetes mellitus [2], which invariably show endothelial dysfunction as well as associated cardiovascular risk factors as hypertension, obesity, and dyslipidemia [3]. Either dyslipidemia, hyperinsulinemia, insulin resistance, or hyperglycemia contributes to the development of endothelial dysfunction [4].

Arterial stiffness, estimated by pulse wave velocity (PWV) determination, has an independent predictive value for cardiovascular events [5], is associated with the severity of coronary artery disease, and is impaired in coronary atherosclerosis [6]. The ambulatory arterial stiffness index (AASI) is related to cardiovascular morbidity-mortality [7] and to the associated target organ damage in hypertensive patients [8]. AASI is very useful for assessing arterial stiffness and is an independent predictor of cardiovascular mortality and morbidity in patients with cardiovascular disease and in healthy individuals. We have previously shown that AASI is positively correlated with carotid intima-media thickness (IMT) and PWV and negatively correlated with glomerular filtration [9]. Another parameter to measure wave reflection and arterial stiffness is the augmentation index (AIx), which is a predictor of adverse cardiovascular events, and higher values are associated with target organ damage [10].

The role of reactive oxygen species (ROS) in the pathophysiology of cardiovascular diseases has been described, as oxidative stress is associated with cardiac and vascular defects leading to hypertension and atherosclerosis [11], but direct cause and effect relationships have not been clearly defined. Although ROS originate from different sources, the vascular NADPH seems to be one of the main sources in cardiovascular pathophysiology [12]. Elevated levels of superoxide anion have been detected in essential hypertension [13] and in the development of atherosclerosis [14]. The enzyme superoxide dismutase (SOD) is an intracellular antioxidant defence mechanism which catalyses the dismutation of superoxide radical into H_2_O_2_ and oxygen [11]. SOD has a protective role in atherogenesis [15] and improves hypertension modulating vasodilation, vasoconstriction, vascular remodelling, and cardiac hypertrophy, playing a relevant role in the development and the maintenance of chronic hypertension in various organs [16].

However, so far there have been no studies that evaluate the possible relationship between serum levels of this enzyme and different vascular parameters with a predictive value on cardiovascular risk. Thus, we have assessed the relationship between SOD serum levels and parameters of vascular function and structure (PWV, AASI, IMT, and AIx) as well as cardiovascular risk in hypertensive and type 2 diabetic patients.

## 2. Materials and Methods

This is a cross-sectional study performed in 307 consecutive patients (54 with type 2 diabetes and hypertension, 16 nonhypertensive diabetic, 185 hypertensive nondiabetic patients, and 52 nondiabetic and nonhypertensive controls), enrolled in the study over a period of 24 months (from January 2008 to January 2010) in the Primary Care Research Unit of La Alamedilla Health Centre (Castilla y León Health Service-SACYL), Salamanca, Spain, which complied with the inclusion/exclusion criteria.

Inclusion Criteria are as follows: patients aged 20–80 years, diagnosed with type 2 diabetes mellitus and/or hypertension. Exclusion criteria are as follows: patients with secondary hypertension, patients unable to comply with the protocol requirements (psychological and/or cognitive disorders, failure to cooperate, educational limitations and problems in understanding written language, and failure to sign the informed consent document), patients participating or who were going to participate in clinical trials during the study, and patients with serious comorbidities representing a threat to life (known coronary or cerebrovascular atherosclerotic disease, heart failure, moderate or severe chronic obstructive pulmonary disease, walking-limiting musculoskeletal disease, advanced respiratory, renal or hepatic disease, severe mental diseases, treated oncological disease diagnosed in the past 5 years, pregnant women, and terminal patients). Most of the patients with hypertension and diabetes received drug therapy (except those controlled by diet), which is described in Table 1.

Sample size calculation indicated that the 307 patients included in the study were sufficient to detect a coefficient correlation of 0.16 between superoxide dismutase with parameters of vascular function and structure in a two-sided test with an alpha risk of 0.05 and a power of 80%.

Hypertension was diagnosed as recommended by The Task Force for the Management of Arterial Hypertension of the European Society of Hypertension and of the European Society of Cardiology [17]. Diabetes was diagnosed as recommended by the Expert Committee on the Diagnosis and Classification of Diabetes Mellitus [18].

### 2.1. Ethical and Legal Issues

The experimental protocol was in accordance with the Declaration of Helsinki (2000) of the World Medical Association and approved by the Ethics Committee of the University Hospital of Salamanca (Spain) and complied with Spanish data protection law 15/1999 and its developed specifications (RD 1720/2007). Each patient signed a participation informed consent after full explanation of the study.

### 2.2. Sociodemographic and Cardiovascular Variables

We evaluated patient age and sex, hypertension, dyslipidemia, alcohol consumption, smoking, history of premature cardiovascular disease (before 55 years of age in males and before 65 in females), and patients on treatment with antihypertensive, antidiabetic, lipid lowering, and antiaggregant drugs.

### 2.3. Serum SOD and 8-Hydroxy-2-deoxyguanosine Determination

Serum concentrations of Cu/Zn SOD and 8-hydroxy-2-deoxyguanosine were determined with ELISA kits (Cu/Zn SOD: Northwest Life Sciences Inc., Vancouver, WA, USA; 8-hydroxy-2-deoxyguanosine: Abcam, Cambridge, UK), according to the instructions of the manufacturer. Absorbance was read on a spectrophotometer (Thermo Luminoskan Ascent, Waltham MA, USA) at 450 nm (Cu/Zn SOD) and 410 (8-hydroxy-2-deoxyguanosine).

### 2.4. Other Biochemical Determinations

Blood samples were collected after patient fasting for at least 8 hours. Determinations are as follows: creatinine, basal glucose, HbA1c, uric acid, HDL-cholesterol, LDL-cholesterol, total cholesterol, and triglycerides. The parameters were measured on a blind basis in a General Hospital Biochemistry laboratory using standard automatized techniques.

### 2.5. Blood Pressure Determination

Office blood pressure evaluation involved three systolic (SBP) and diastolic blood pressure (DBP) measurements, using the average of the last two measurements, with a validated OMRON model M10-IT sphygmomanometer (Omron Health Care, Kyoto, Japan), following the recommendations of the European Society of Hypertension [19]. Pulse pressure was estimated with the mean values of the second and third measurements.

Ambulatory blood pressure monitoring (ABPM) was performed on a day of standard activity using a control system (Spacelabs 90207, Healthcare, Issaquah, Washington, USA). We obtained blood pressure measurements every 20 min (waking period) and every 30 min (resting period). Valid records of readings were 80% of the total.

### 2.6. Determination of Pulse Wave Velocity (PWV) and Peripheral (PAIx) and Central (CAIx) Augmentation Index

These parameters were estimated using the SphygmoCor System (AtCor Medical Pty Ltd., Head Office, West Ryde, Australia). Pulse wave analysis was performed with a sensor in the radial artery, using mathematical transformations to estimate the aortic pulse wave. CAIx was estimated from the morphology of the aortic wave using the following formula: increase in central pressure × 100/pulse pressure. PAIx was calculated as follows: (second peak SBP [SBP2] − [DBP])/(first peak SBP − DBP) × 100 (%). Measurements reliability was evaluated using the CAIx intraclass correlation coefficient, which showed values of 0.974 (95% CI: 0.936–0.989) for intraobserver agreement on repeated measurements in 22 subjects. According to the Bland-Altman analysis, the limit of intraobserver agreement was 0.454 (95% CI: −9.876–10.785). The carotid-femoral pulse wave was analysed estimating the delay with respect to the ECG wave and calculating the PWV. Distance measurements were taken with a measuring tape from the sternal notch to the carotid and femoral arteries at the sensor location.

### 2.7. Ambulatory Arterial Stiffness Index (AASI)

Arterial stiffness was evaluated with AASI, defined as one minus the regression slope of DBP over SBP readings obtained from individual 24-hour blood pressure recordings. The stiffer the arterial tree, the closer the regression slope and AASI to 0 and 1, respectively.

### 2.8. Assessment of Carotid Intimamedia Thickness (C-IMT)

Carotid ultrasound to assess carotid IMT was performed by two investigators trained for this purpose before starting the study. The reliability of such recordings was evaluated before the study, using the intraclass correlation coefficient, which showed values of 0.97 (95% CI: 0.94 to 0.99) for intraobserver agreement on repeated measurements in 20 subjects, and 0.90 (95% CI: 0.74 to 0.96) for interobserver agreement. According to the Bland-Altman analysis, the limit of interobserver agreement was 0.02 (95% limits of agreement: −0.05–0.10), and the limit of intraobserver agreement was 0.01 (95%: −0.03–0.06). A Sonosite Micromax ultrasound (Sonosite Inc., Bothell, Washington, USA) device paired with a 5–10 MHz multifrequency high-resolution linear transducer with Sonocal software was used for automatic measurements of IMT to optimize reproducibility. Measurements were made of the common carotid after the examination of a longitudinal section of 10 mm at a distance of 1 cm from the bifurcation, performing measurements in the proximal wall and in the distal wall in the lateral, anterior, and posterior projections, following an axis perpendicular to the artery to discriminate two lines: one for the intima-blood interface and the other for the media-adventitious interface. 6 measurements were obtained in both the right and the left carotid, using average values (average C-IMT) and maximum values (maximum C-IMT) automatically calculated by the software [20]. The average IMT was considered abnormal if it measured 0.90 mm, or if there were atherosclerotic plaques with a diameter of 1.5 mm or a focal increase of 0.5 mm or 50% of the adjacent IMT [17].

### 2.9. Cardiovascular Risk Assessment

Risk of cardiovascular morbidity and mortality was estimated using the risk equation (D'Agostino scale) based on the Framingham study [21]. The individuals performing the different tests were blinded to the clinical data of the patient. All organ damage assessment measures were made within a period of 10 days.

### 2.10. Statistical Analysis

**Figure 1:**
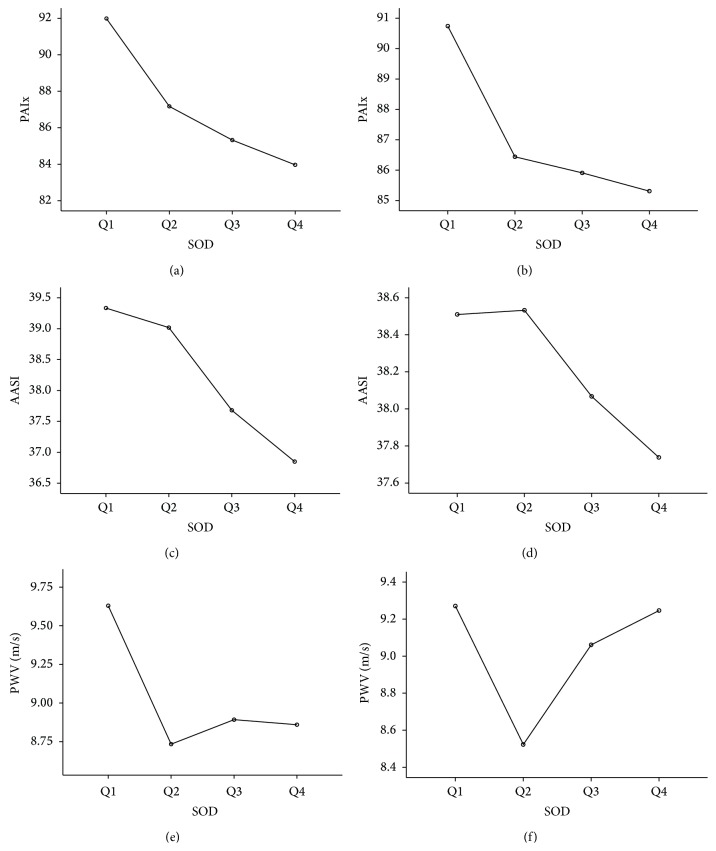
Peripheral augmentation index, ambulatory arterial stiffness index, and pulse wave velocity values divided into 4 quartiles according to SOD serum levels. Multivariate analysis: AASI, PAIx, PWV, and IMT are dependent variables, and SOD is the independent variable. (b), (d), and (f) show values after adjusting for age the independent variable. AASI: ambulatory arterial stiffness index; PAIx: peripheral augmentation index; PWV: pulse wave velocity. *p* values: (a): 0.166; (b): 0.469; (c): 0.076; (d): 0.825; (e): 0.102; (f): 0.090.

Data input was made using the Teleform system (Autonomy Cardiff, Vista, CA, USA), exporting the data to the PASW version 18.0 statistical package (SPSS Inc., Chicago IL, USA). Data was presented as mean ± standard deviation or percentage. One-way analysis of variance (ANOVA) for independent samples was used to compare quantitative variables among SOD quartiles. Pearson correlation test was used to analyze associations between quantitative variables. Using the general linear model procedure, we have conducted two multivariate analyses in which we have considered AASI, PAIx, PWV, and IMT as dependent variables, and SOD quartiles as independent variables. We have performed a first model without adjustments and a second model adjusted for age in each of the independent variables (represented in Figure 1). Hypothesis contrasting established an alpha risk factor of 0.05 as the limit of statistical significance.

## 3. Results 

General and medical characteristics of the patients are presented in Table 1. The average age of the patients was 55 years, and 62 percent of them were male. 143 subjects (46.6%) have antihypertensive treatment, 66 (21.5%) with antidiabetic treatment, and 84 (27.4%) with lipid-lowering therapy, of whom 74 (24%) are taking statins, 7 (2.3%) are taking fibrates, and 6 (2%) other lipid-lowering drugs. The values of the different parameters of vascular structure and function (IMT, PWV, PAIx, CAIx, and AASI) are lower in the control group and higher in the group with diabetes and hypertension associated (*p* < 0.001) (Table 1). Plasma levels of 8-hydroxy-2-deoxyguanosine, one of the predominant forms of free radical-induced oxidative lesions, were similar in the different groups of patients.

In our study population, serum SOD was inversely correlated with PWV, PAIx, CAIx, AASI, and pulse pressure. We also found a negative correlation with plasma HDL-cholesterol and a positive correlation with uric acid levels and triglycerides only in hypertensive patients, correlations that remain after adjusting the data for the intake of antihypertensive and lipid-lowering drugs (Table 2).

After dividing the sample into quartiles according to SOD serum levels, the values of PAIx, AASI, PWV, and cardiovascular risk estimated with D'Agostino index are higher in the first quartile, although only AASI shows statistically significant differences (*p* = 0.019) (Table 3). This trend was maintained after adjusting for age in the case of PAIx and AASI (Figure 1).

## 4. Discussion 

This is the first study linking serum SOD levels with PWV, AIx, AASI, and pulse pressure, thus suggesting that oxidative stress significantly affects the vascular structure and function in hypertensive and diabetic patients and showing the role of SOD as an indicator of hypertension and diabetes-induced impairment of cardiovascular function, cardiovascular risk, and target organ damage. After adjusting for age, we still detect correlations between SOD and PAIx, AASI, and IMT, confirming the role of this serum marker as predictor of alterations in vascular structure and function.

Serum levels of SOD are negatively correlated with PWV, PAIX, CAIx, and AASI in our study population (hypertensive and diabetic patients recruited from primary care centers), thus suggesting that oxidative stress promotes or encourages the development of endothelial dysfunction, as it has been previously suggested by other authors [22]. Although the role of CAIx in the clinical setting remains unclear, it seems to be a predictor of adverse cardiovascular events in several patient populations, and higher AIx is associated with target organ damage [10]. According to our data, it has been previously observed that SOD improves endothelial function [23] and Mn-SOD protects against oxidative stress and endothelial dysfunction in ApoE-deficient mice [24].

We detected negative correlations between serum SOD and most of the parameters of vascular structure and function analysed. Our data suggest that serum SOD is not increased in response to vascular injury in hypertensive and diabetics patients; its serum levels are not increased in these patients with vascular disorders, but its decline indicates a deficit in antioxidant defence mechanisms, since hypertensive and diabetic patients are unable to remove the circulating superoxide anion and therefore suffer an increase in vascular damage induced by reactive oxygen species. Thus, a lower level of serum SOD is associated with increased vascular damage.

We observed a negative correlation between SOD and HDL-cholesterol and positive correlation between SOD and triglycerides. HDL-cholesterol levels are inversely related to the risk of clinical events due to atherosclerosis [23]. On the other hand, a univariate association between triglycerides and cardiovascular risk has been described [25]. However, although the relationship between SOD serum levels with HDL-cholesterol and triglycerides has not been previously described, our results suggest that these molecules are related but further experiments are needed to assess the nature and characteristics of this relationship.

In our study population, there are positive correlations between SOD and uric acid. In agreement with our finding, Brand et al. [26] described a strong correlation between hyperuricemia and elevated cardiovascular risk in the general population, and it has been observed that serum uric acid correlates with extracellular SOD activity in patients with chronic heart failure [27]. As we said previously regarding HDL-cholesterol and triglycerides, additional experiments are needed to elucidate the pathophysiological significance of this correlation.

Limitations of our study are its cross-sectional design, which precludes longitudinal analysis between SOD, vascular structure, and function. We have analysed SOD expression level, but not its activity. Sampling of the study was performed consecutively, including hypertensive patients with a short course of hypertension, or with diabetes and hyperlipidemia, and many patients receiving drug therapy, which may modify blood pressure levels. However, the heterogeneity of the sample is similar to the distribution of the real population of short-course hypertensive patients with some risk factors and without previous cardiovascular disease.

## 5. Conclusions 

We describe for the first time that SOD serum levels are correlated with alterations in vascular structure and function, being an indicator of cardiovascular alterations, cardiovascular risk, and target organ damage in hypertensive and diabetic patients, thus confirming that oxidative stress negatively contributes to the proper functioning of blood vessels. These encouraging data have to be followed by prospective studies to establish the relative strength of the prediction of cardiovascular risk and the appearance of target organ damage according to the SOD levels presented by the patient.

## Figures and Tables

**Table 1:** Characteristics of study population.

	All patients	DIA + HYP	DIA	HYP	Control	*p*
Number	307	54	16	185	52	
Age (years)	54.76 ± 11.69	60.75 ± 8.35	55.27 ± 12.82	55.26 ± 10.99	46.63 ± 12.54	<0.001
Male sex (*N*, %)	191 (62.21%)	39 (72.22%)	11 (68.75%)	108 (58.38%)	33 (63.46%)	0.286
Superoxide dismutase (ng/mL)	123.48 ± 57.20	134.76 ± 63.60	112.00 ± 35.23	117.63 ± 47.69	137.91 ± 82.68	0.079
8-Hydroxy-2-deoxyguanosine (ng/mL)	6.97 ± 5.07	7.97 ± 5.52	6.24 ± 4.17	6.35 ± 5.13	7.40 ± 4.76	0.377
Systolic blood pressure (mmHg)	138.26 ± 17.15	142.59 ± 18.20	119.31 ± 9.61	143.66 ± 16.09	126.26 ± 8.92	<0.001
Diastolic blood pressure (mmHg)	86.89 ± 10.92	84.94 ± 12.07	74.13 ± 5.19	90.62 ± 9.88	79.56 ± 6.66	<0.001
Pulse pressure (mmHg)	52.49 ± 12.37	57.37 ± 12.92	45.39 ± 10.15	53.16 ± 12.23	46.91 ± 10.00	<0.001
IMT medium average (mm)	0.73 ± 0.11	0.80 ± 0.11	0.73 ± 0.12	0.73 ± 0.10	0.68 ± 0.14	<0.001
IMT maximum average (mm)	0.90 ± 0.14	0.98 ± 0.12	0.90 ± 0.15	0.90 ± 0.12	0.84 ± 0.17	<0.001
Pulse wave velocity (m/s)	8.98 ± 2.22	10.39 ± 2.35	8.65 ± 2.27	9.00 ± 2.09	7.59 ± 1.61	<0.001
Peripheral augmentation index	92.76 ± 21.43	95.85 ± 20.87	88.94 ± 22.34	95.15 ± 21.45	82.22 ± 18.63	0.001
Central augmentation index	30.31 ± 11.66	31.35 ± 10.67	30.44 ± 12.10	31.81 ± 10.94	23.85 ± 13.04	<0.001
AASI	38.25 ± 6.05	41.39 ± 5.96	38.27 ± 5.44	37.71 ± 5.89	36.92 ± 5.96	<0.001
D'Agostino cardiovascular risk	19.73 ± 16.27	36.59 ± 18.22	17.34 ± 11.88	18.00 ± 13.72	8.59 ± 8.24	<0.001
Smokers (*N*, %)	73 (23.77%)	13 (24.07%)	4 (25%)	38 (20.54%)	18 (34.62%)	0.202
HDL (mg/dL)	51.98 ± 12.45	47.48 ± 9.73	50.75 ± 12.68	52.86 ± 12.63	53.96 ± 13.42	0.023
LDL (mg/dL)	125.91 ± 32.76	108.13 ± 27.96	108.50 ± 22.07	132.71 ± 34.03	125.75 ± 26.68	<0.001
Total cholesterol (mg/dL)	204.40 ± 36.98	187.46 ± 34.70	183.81 ± 23.66	211.44 ± 37.58	203.25 ± 32.77	<0.001
Alcohol consumption (units/week)	10.98 ± 20.28	14.94 ± 21.35	3.69 ± 4.87	9.63 ± 15.81	13.87 ± 32.15	0.111
HbA1c (%)	5.41 ± 1.05	6.84 ± 1.40	6.68 ± 0.80	5.05 ± 0.46	4.83 ± 0.40	<0.001
Glycemia (mg/dL)	98.44 ± 30.44	136.61 ± 46.27	132.63 ± 32.63	88.63 ± 11.14	83.19 ± 8.75	<0.001
Antihypertensive drugs (*N*, %)	143 (46.58%)	48 (88.89%)	0 (0.00%)	95 (51.35%)	0 (0.00%)	<0.001
Antidiabetic drugs (*N*, %)	66 (21.50%)	51 (94.44%)	15 (93.75%)	0 (0.00%)	0 (0.00%)	<0.001
Lipid-lowering drugs (*N*, %)	84 (27.36%)	32 (59.26%)	6 (37.50%)	43 (23.24%)	3 (5.77%)	<0.001
Antiaggregants (*N*, %)	61 (19.87%)	29 (53.70%)	6 (37.50%)	23 (12.43%)	3 (5.77%)	<0.001

Demographic, physical, and medical characteristics and drug therapies of patients included in the study. Data are expressed as mean ± standard deviation or percentage. AASI: ambulatory arterial stiffness index; DIA: diabetic nonhypertensive patients; DIA + HYP: diabetic hypertensive patients; HbA1c: glycosylated haemoglobin; HDL: cholesterol associated with high density lipoproteins; HYP: hypertensive patients; IMT: intima-media thickness; LDL: cholesterol associated with low density lipoproteins; *p*: *p* value, statistically significant differences (ANOVA).

**Table 2:** Pearson correlations between serum superoxide dismutase and parameters of vascular structure and function and cardiovascular risk.

	Superoxide dismutase				
	All patients	DIA + HYP	DIA	HYP	Control
Intima-media thickness medium average	−0.08	0.06	0.45	−0.12	−0.22
Intima-media thickness maximum average	−0.06	0.06	0.41	−0.07	−0.22
Pulse wave velocity	−0.15^*∗*^	−0.06	−0.03	−0.16^*∗*^	0.25
Peripheral augmentation index	−0.16^*∗∗*^	−0.08	−0.09	−0.13	−0.37^*∗*^
Central augmentation index	−0.16^*∗∗*^	−0.01	−0.18	−0.10	−0.35^*∗*^
AASI	−0.19^*∗∗*^	−0.32^*∗*^	0.20	−0.24^*∗∗*^	−0.15
Pulse pressure	−0.17^*∗∗*^	−0.20	−0.07	−0.18^*∗*^	−0.19
D'Agostino cardiovascular risk	−0.03	−0.09	0.46	−0.06	−0.10
8-Hydroxy-2-deoxyguanosine	−0.17	−0.29	0.21	−0.21	−0.22
Uric acid	0.19^*∗∗*^	0.19	0.25	0.20^*∗∗*^	0.28
HDL	−0.18^*∗∗*^	−0.10	−0.22	−0.18^*∗*^	−0.28
LDL	0.02	−0.06	0.40	0.13	−0.10
Triglycerides	0.19^*∗∗*^	0.19	0.44	0.20^*∗∗*^	0.25

AASI: ambulatory arterial stiffness index; DIA: diabetic nonhypertensive patients; DIA + HYP: diabetic hypertensive patients; HDL: cholesterol associated with high density lipoproteins; HYP: hypertensive patients; LDL: cholesterol associated with low density lipoproteins. Statistical significant differences: ^*∗*^ *p* < 0.05; ^*∗∗*^ *p* < 0.01.

**Table 3:** Parameters of vascular structure and function divided into 4 quartiles according to superoxide dismutase serum levels.

	Q1 (SOD: <87.3 ng/mL)	Q2 (SOD: 87.3–112.8 ng/mL)	Q3 (SOD: 112.8–142.6 ng/mL)	Q4 (SOD: >142.6 ng/mL)	*p*
IMT medium average (mm)	0.73 ± 0.11	0.75 ± 0.13	0.73 ± 0.10	0.72 ± 0.12	0.436
IMT maximum average (mm)	0.90 ± 0.12	0.92 ± 0.16	0.90 ± 0.12	0.89 ± 0.14	0.620
PWV (m/s)	9.64 ± 2.47	8.83 ± 1.94	8.83 ± 2.46	8.88 ± 1.81	0.088
Central AIx	29.41 ± 9.87	30.72 ± 9.50	27.04 ± 10.67	26.77 ± 10.94	0.076
Peripheral AIx	91.92 ± 22.84	87.72 ± 14.71	85.32 ± 22.48	84.05 ± 22.82	0.167
AASI	39.62 ± 6.40	39.29 ± 6.69	37.64 ± 5.84	36.74 ± 5.34	0.019
D'Agostino cardiovascular risk	21.75 ± 19.30	18.30 ± 13.79	19.92 ± 17.11	19.91 ± 14.79	0.705

Data are expressed as mean ± standard deviation. AASI: ambulatory arterial stiffness index; AIx: augmentation index; CV cardiovascular; IMT: intima-media thickness; *p*: *p* value, statistically significant differences (ANOVA); PWV: pulse wave velocity.

## Abbreviations

AASI: Ambulatory arterial stiffness index
ACEi: Angiotensin converting enzyme inhibitors
AIx: Augmentation index
ARB: Angiotensin receptor blockers
CV: Cardiovascular
DIA: Diabetic nonhypertensive patients
DIA + HYP: Diabetic hypertensive patients
HbA1c: Glycosylated haemoglobin
HDL: Cholesterol associated with high density lipoproteins
HYP: Hypertensive patients
IMT: Intima-media thickness
LDL: Cholesterol associated with low density lipoproteins
PWV: Pulse wave velocity
SOD: Superoxide dismutase.
